# RE: Are Cross-Sectional Imaging Modalities Enough for Sarcopenia Assessment?

**DOI:** 10.5152/tjg.2024.234252

**Published:** 2024-01-01

**Authors:** Rasim Eren Cankurtaran, Yasin Celal Güneş, Emre Dirican, Oktay Algın, Damla Cankurtaran, Öykü Tayfur Yürekli

**Affiliations:** 1 Department of Gastroenterology, Ankara Yıldırım Beyazıt University Faculty of Medicine, Ankara, Turkey; 2 Department of Radiology, Ankara Atatürk Sanatorium Training and Research Hospital, Ankara, Turkey; 3 Department of Medical Informatics and Biostatistics, Mustafa Kemal University Faculty of Medicine, Hatay, Turkey; 4 Department of Radiology, Ankara University Faculty of Medicine, Ankara, Turkey; 5 Department of Physical Medicine and Rehabilitation, University of Health of Sciences Dışkapı Education and Research Hospital, Ankara, Turkey

Dear editor,

We would like to express our gratitude to Kani and Tufan^1^ for taking the time to provide feedback on our article^2^ published in Turkish Journal of Gastroenterology on June 27, 2023. We appreciate your thoughtful comments and insights, which have added value to our research.

We acknowledge and agree with their points regarding the limitations of our study in measuring sarcopenia and its potential impact on the comprehensiveness of our findings. The recommendations from the European Working Group on Sarcopenia in Older People (EWGSOP) to use hand-grip strength and various radiological methods for muscle strength and mass assessment are indeed valuable and align with the evolving understanding of sarcopenia. However, I would like to emphasize that our study is retrospective in nature, and as a result, it was not feasible for us to measure muscle strength at the time of cross-sectional imaging.

In their criticisms related to the results of our study, we would like to highlight a significant finding that the presence of sarcopenia is an independent risk factor for surgery, as evident in the multivariate analysis.

Their feedback underscores the importance of collaboration and dialogue within the scientific community to improve the quality and accuracy of our research. We appreciate their commitment to advancing the field of sarcopenia and look forward to potential collaborations or discussions in the future.

Once again, we would like to thank them for their valuable insights, and we would like to let them know to feel free to reach out if they have any further comments or suggestions. We value their expertise and perspective.
